# LONGEVITY DETERMINANTS IN AFRICAN AMERICANS: AN INTRA-GROUP ANALYSIS FROM THE HEALTH AND RETIREMENT STUDY

**DOI:** 10.1093/geroni/igad104.3455

**Published:** 2023-12-21

**Authors:** Meneka Johnson Nicholson, Peter Martin

**Affiliations:** Rural Health Medical Program, Inc., Selma, Alabama, United States; Iowa State University, Ames, Iowa, United States

## Abstract

Utilizing the Health and Retirement Study data, this research exclusively centered on a sample of 2953 African-American participants to discern longevity predictors, emphasizing the importance of intra-group analysis. This targeted approach is essential as it provides a nuanced understanding of factors affecting longevity within a specific demographic group, avoiding broad generalizations derived from diverse populations. Two analytical models were developed in Mplus. The initial fully saturated model was subsequently trimmed to exclude non-significant paths. Key findings include: Education showed a positive correlation with cognition and a negative association with subjective health. Age had a negative impact on cognition and on subjective health. Depression was negatively associated with cognition and subjective health, and positively with age. Notably, age at death was majorly influenced by age, with subjective health, cognition, and education also playing significant roles. The model demonstrated an exemplary fit with Chi^2 (df=2) = 3.12, RMSEA = .01, and CFI = 1.0. Ultimately, age was identified as the primary predictor of age at death. Moreover, subjective health, education, and cognitive abilities emerged as vital contributors to longevity among African Americans. In essence, this study, with its focus on African Americans, underscores the profound importance of demographic-centric research, charting a path for custom-made interventions that resonate deeply with the African-American community’s inherent health dynamics.

